# “Making the Child Mine”: Mothers’ Thoughts and Feelings About the Mother–Infant Relationship in Egg Donation Families

**DOI:** 10.1037/fam0000619

**Published:** 2020-01-16

**Authors:** Susan Imrie, Vasanti Jadva, Susan Golombok

**Affiliations:** 1 Centre for Family Research, University of Cambridge

**Keywords:** egg donation, assisted reproductive technology, gamete donation, infancy, mother–infant relationship

## Abstract

The number of families being created through fertility treatment with donor eggs is increasing yearly. Women who conceive in this way share a gestational but not genetic relationship with their child, yet there is limited understanding of how mothers experience the mother–child relationship during its formative period, infancy. This study explored heterosexual mothers’ thoughts and feelings about the mother–infant relationship in families created through egg donation. Qualitative interviews were conducted with a sample of 85 women who had conceived following egg donation treatment at U.K. fertility clinics. Mothers had at least 1 infant (6–18 months) and were living with the child’s father. Interview data were analyzed according to the principles of thematic analysis. The results showed that egg donation mothers used a range of strategies across the transition to parenthood that enabled them to establish their identity as the child’s mother and facilitated the process of helping them feel that the baby was their own. This process was individual to each woman, with the absent genetic connection varying in significance between mothers. The strategies employed enabled most mothers to adjust successfully to parenthood and manage any ambivalence and uncertainties associated with nongenetic parenthood. Most mothers felt secure and confident in their position as the child’s mother by the end of the first year.

The quality of the relationship that forms between a parent and their infant has long been identified as influencing a range of child outcomes (Fearon, Bakermans-Kranenburg, van IJzendoorn, Lapsley, & Roisman, 2010; Madigan, Atkinson, Laurin, & Benoit, 2013). While a substantial body of empirical work has addressed the ways in which individual differences in parent–infant relationship quality predict later outcomes, less work has addressed parents’ thoughts and feelings about the developing relationship and their perceptions of the process through which these relationships form (Figueiredo, Costa, Pacheco, & Pais, 2007). Even less is known about how parents in diverse family forms, such as those created through assisted reproductive technologies, experience their early parent–infant relationships. This has been highlighted as an area deserving of attention (Goldberg, Moyer, & Kinkler, 2013) as diverse family forms grow in both number and visibility (Golombok, 2015). The present study aimed to examine heterosexual mothers’ experiences of the mother–infant relationship in families created through egg donation, an increasingly common method of family building (Human Fertilisation and Embryology Authority [HFEA], 2019b), in which mothers and their children do not share a genetic relationship.

First proposed by attachment theorists, a growing body of research has examined the ways in which parents’ representations—that is, their thoughts and feelings—about their infant guide their behavior with the child (George & Solomon, 1996), and highlights the importance of understanding parents’ representations of their child prenatally (Foley & Hughes, 2018) and in the first year of life (Vreeswijk, Maas, & van Bakel, 2012). Mothers’ prenatal representations of their infant have been found to be associated with later mother–child interaction quality (Foley & Hughes, 2018), as have their representations during the first year (Korja et al., 2010). Similarly, the related literature on parental bonding emphasizes that the bonding process includes a mental component (de Cock et al., 2016), begins during pregnancy and continues beyond the early postnatal period (Klaus, Kennell, & Klaus, 1995), and is related to child outcomes (Mason, Briggs, & Silver, 2011). It seems crucial, then, that any assessment of parent–infant relationship quality should consider parents’ representations of the parent–infant relationship (Vreeswijk et al., 2012).

Studies of early parent–infant relationships have highlighted the variability in the formation of parents’ affectional bonds with their babies (Figueiredo et al., 2007). This variability has also been found in adoptive parents’ perceptions of the parent–child bond (Goldberg et al., 2013). The transition to adoptive parenthood literature indicates that parents who do not have a genetic relationship with their child face additional challenges during the transition to parenthood, which may or may not affect their feelings about the formation of these affectional bonds (Goldberg et al., 2013). While adoptive parenthood and egg donation parenthood differ in several key ways, in that mothers through egg donation gestate and give birth to their children, the psychological literature on adoptive parenthood does highlight some of the challenges and complexities involved for parents considering and pursuing nongenetic parenthood. Mothers in both family types challenge dominant cultural discourses that both assume a genetic connection between mothers and their children (Kirkman, 2008), and prioritize these over social relatedness in the creation and maintenance of kin relationships (Freeman, 2014). Both have also reported awareness of stigma around nongenetic parenthood and their “nontraditional” path to parenthood (Goldberg, Kinkler, & Hines, 2011; Golombok et al., 2004), with suggestions that women may perceive higher levels of stigma about nongenetic parenthood than men (Goldberg et al., 2011). Both family types, in the case of heterosexual couple families, also typically pursue these paths to parenthood following infertility.

Additionally, both family types may face specific challenges around parental identity. Kirk’s (1964) seminal text on adoption highlighted that new parents may face uncertainty around their parental role definition and that they employed a “rejection of difference” strategy (involving enacting beliefs and behaviors that reject or deny the difference of being an adoptive parent), and/or an “acknowledgment of difference” strategy (which involved actively confronting any challenges around adoptive parenthood and allowing for the exploration of ambivalent feelings associated with being different), in order to manage these challenges. More recent empirical work showed that adoptive parents continue to face concerns about their sense of emotional entitlement to parent a genetically unrelated child (Timm, Mooradian, & Hock, 2011), a process which may take months (Timm et al., 2011), and raise concerns about whether they will feel able to love a genetically unrelated child (Daniluk & Hurtig-Mitchell, 2003). It has been suggested that pursuing nongenetic motherhood within this context, through adoption, requires women to reconfigure their conceptualizations of parenthood (Goldberg, Downing, & Richardson, 2009) and address the loss of the imagined genetically related child (Daniluk & Hurtig-Mitchell, 2003).

Sandelowski, Harris, and Holditch-Davis (1993) identified a process they termed “parental claiming,” which described intellectual and emotional work undertaken by prospective parents in order to gain a sense of entitlement to parent a genetically unrelated child (Sandelowski et al., 1993). This process began when couples first considered adoption and was ongoing, involving undermining the primacy of genetic ties. Couples also used examples of close, nongenetic relationships to provide an experiential framework that allowed them to establish that genetic ties were neither essential criteria in determining parental status nor necessary for establishing and maintaining a loving relationship. Whether mothers through egg donation undergo a similar process of parental claiming is not known.

Research on families created through egg donation has tended to focus on quantitative assessments of family functioning (Golombok et al., 2004, 2006, 2011; Golombok, Blake, Casey, Roman, & Jadva, 2013; Golombok, Ilioi, Blake, Roman, & Jadva 2017; Golombok, Jadva, Lycett, Murray, & MacCallum, 2005; Imrie, Jadva, Fishel, & Golombok, 2019), or parents’ decisions about whether to disclose the method of conception to the child (Readings, Blake, Casey, Jadva, & Golombok, 2011; Söderström-Anttila, Sälevaara, & Suikkari, 2010). Little is known about new egg donation parents’ thoughts and feelings about egg donation parenthood and even less about the way in which the mother–child relationship is experienced during its formative period, infancy.

Research examining the mother–infant relationship in egg donation families has tended to rely on survey data, small, heterogeneous samples, or retrospective accounts of the mother–infant relationship. Furthermore, as some professional bodies move toward encouraging parents to disclose donor conception to their children, the sociocultural landscape around egg donation may differ from that experienced by parents in previous decades (Ethics Committee of the American Society for Reproductive Medicine, 2018; HFEA, 2019a). A Dutch survey of egg donation mothers who had had children over a 12-year period found that over 80% had thought about nongenetic motherhood during pregnancy, but two thirds reported that egg donation had not influenced the mother–child relationship (Van Berkel, Candido, & Pijffers, 2007). Conversely, a survey of women who had conceived through egg donation in the previous 5 years in Spain, of whom 48% had traveled from England, found that only 4% reported that they had felt concerned prior to birth about bonding with their baby, and nearly half stated that they had not felt concerned (Hertz & Nelson, 2016). Many viewed the absence of a genetic connection as of little significance, with some reporting instantaneous bonding with their baby as soon as they learned they were pregnant. Similarly, a survey of American mothers found that women reported feeling an emotional connection in utero and instant bonding with their infant at birth (Applegarth & Riddle, 2007). However, as details of the method of data analysis used were not provided, how representative of the sample this statement is remains unclear.

Qualitative studies have provided a more nuanced understanding of mothers’ representations of the mother–infant relationship, with a small study of four British mothers with infants finding that women reported concerns during pregnancy about whether they would feel like the child’s “real mother” (Stuart-Smith, Smith, & Scott, 2012). Once the baby was born, however, these fears were not realized; the process of being pregnant, and the ease with which they had bonded with their newborn contributed to their sense of identity as the baby’s mother (Stuart-Smith et al., 2012).

Kirkman’s (2008) qualitative study of 19 women who had between one and four egg donation children highlighted the ambivalent and complex feelings experienced by some mothers. Some women interpreted their egg donation motherhood as no different to genetic motherhood, and several maintained a “mystical sense” that the process of gestating the child conferred something akin to a genetic link. Yet others reported that feelings of not being “real” mothers persisted and that these feelings were rooted partly in the lack of genetic relationship with their child. For some mothers this remained a “meaningful absence,” and the gestational connection, although providing some comfort, could not entirely overcome this. She also found that as the women enacted motherhood in their day-to-day encounters with their children, they emphasized the importance of nurturance over genetic relatedness in legitimating motherhood (Kirkman, 2008).

Although this small body of research has started to explore egg donation mothers’ feelings about their relationship with their children, few studies were designed to explicitly address this question. As yet, no study has investigated egg donation mothers’ thoughts and feelings about their relationship with their infant, and the significance (or lack thereof) attributed to egg donation in the developing mother–infant relationship. The present study aimed to address these questions through qualitative interviews with a sample of heterosexual egg donation mothers with infants. Given the importance of infancy as a period in which parent–child relationship are initially formed, and the growing use of egg donation as a family-building option, an exploration of how women navigate, understand, and experience parenting a child conceived in this way seems timely.

## Method

### Sample Characteristics

Participants were recruited through 12 fertility clinics in the United Kingdom. Mothers had at least one child aged 6–18 months conceived through egg donation and lived with the child’s father. The inclusion criteria were determined by mothers’ involvement in a larger study of family functioning in families created through IVF (Imrie et al., 2019).

All families were initially contacted by letters from clinics and invited to send their contact details to the researchers if they were interested in learning more about the study. Letters were sent to 275 egg donation families and 110 returned their contact details to the research team (response rate = 40%). Of the 110 families, 99 were eligible for the study, and 85 agreed to take part (participation rate = 86%). Mothers were aged between 33 and 52 years (*M* = 42.45, *SD* = 4.27). Most had one child (*n* = 60, 71%) and most had undertaken higher education (*n* = 55, 73%). Seventy-three (86%) mothers had used identity-release egg donation (where the donor is unknown to the recipient but the resultant child can access identifiable donor information at age 18), and 12 (14%) had used a known donor (where the donor was known to the recipient, e.g., a sister). The mean age of the infants was 11 months (*SD* = 2.15).

### Interviews and Analysis

Each mother took part in a qualitative interview conducted by one of two researchers in the family home. The first section of the interview comprised the Parent Development Interview (PDI; Aber, Slade, Berger, Bresgi, & Kaplan, 1985; Slade, Belsky, Aber, & Phelps, 1999). The PDI is derived from attachment theory and is a semistructured interview that explores parents’ representations of the parent–child relationship and is based on the idea that parents’ thoughts and feelings about their child influence their parenting. Parents are asked to describe their own and their child’s experiences in moments of relatedness and interaction. The PDI has been used previously with both egg donation (Golombok et al., 2005) and adoptive samples (Steele et al., 2008). The second section of the interview related to experiences of fertility treatment and egg donation. Participants were asked about their fertility treatment history (including how long they had been trying to conceive, their reasons for using fertility treatment, and the type[s] of treatment attempted), and their experiences of egg donation (including the type of egg donation chosen, reasons for, and thoughts about, this and their feelings prior to treatment). The format of these questions was drawn partly from previous studies of egg donation parents (Golombok et al., 2004), and partly from the existing egg donation literature. Both sections of the interview were semistructured, allowing interviewers to include probes where necessary. All interviews were audio recorded. Both sections of the interviews were transcribed verbatim, pseudonyms were used to protect participants’ anonymity, and all identifying information was removed from the transcripts. Interview transcripts varied in length (3,837–14,143 words).

Data were analyzed according to the principles of thematic analysis described by Braun and Clarke (2006) and thematic networks (Attride-Stirling, 2001). Data analysis was carried out by the first author. The analyses followed an inductive, data-driven approach, in that initial analyses of the dataset were undertaken without the use of a predetermined coding scheme, meaning that the themes identified are strongly linked to the data themselves. The analysis consisted of a systematic, phased process, including familiarization with the data where all transcripts were read and reread, memo writing, and coding. All parts of participants’ talk (from both sections of the interview) that discussed the mother–infant relationship or experiences of egg donation were coded. After initial coding, all coded data extracts were collated, and codes were sorted into themes. Themes were assessed for internal homogeneity and external heterogeneity and were refined to produce one global theme, eight organizing themes, and five basic themes (Attride-Stirling, 2001). Peer debriefing was undertaken during the process of theme refinement to ensure that the final thematic map was reflective of the dataset in its entirety.

Gaskell and Bauer’s (2000) two broad criteria of confidence and relevance were adhered to in the design of the study, data collection, data analysis and presentation of the results in order to ensure the quality of the research. Confidence and relevance criteria are based on the principles of evidence-based claims-making and public accountability. Confidence markers “allow the reader and receiver of research to be ‘confident’ that the results of the research represent ‘reality’” (Gaskell & Bauer, 2000, p.344), and relevance markers scrutinize “the import of the research evidence for the people involved, for the theory or concepts at stake, or for the purposes of the research project” (Gaskell & Bauer, 2000, p.363).

Written informed consent was obtained from all study participants, and ethical approval was obtained from the University of Cambridge Psychology Research Ethics Committee.

## Results

Egg donation mothers’ representations of their relationship with their infant, and the significance attributed to egg donation in the mother–infant relationship, could be understood in relation to one global theme “making the child mine” and eight organizing themes: (a) preconception strategies, (b) importance of pregnancy, (c) establishing a bond, (d) child as wanted, (e) mother’s influence beyond genetics, (f) physical resemblance, (g) managing reminders of egg donation, and (h) finding a place for the donor. Two of the organizing themes (preconception strategies and managing reminders of egg donation) comprised several basic themes. The theme “preconception strategies” comprised (a) decisions about donation type, (b) minimization of the donor’s contribution, and (c) normalization of family diversity, and “managing reminders of egg donation” comprised (a) comments about the child’s appearance, and (b) other reminders. All themes are described in detail below, with illustrative quotations throughout.

### Making the Child Mine

Egg donation mothers used a variety of strategies that appeared to facilitate the process of helping them to feel that the baby was their own, in other words “making the child mine.” This was an ongoing process, which began before conception and continued throughout infancy, with strategies used to greater or lesser extents by different women.

For most mothers the strategies were successful, in that they consistently reported that the infant was “my” child and “feels like mine.” Many women emphasized that they felt they could not love their baby more, “I don’t think I could love him any more if he was completely my genetic material” (Grace). A small number of women, however, described still struggling with the idea that their child was not genetically related to them, “Biologically they’re not my children, even though I was pregnant with them and I gave birth to them, and that’s still quite a hard thing for me to square in my own mind” (Wendy). For several mothers this was expressed in terms of an ongoing, although not overwhelming, sadness when they thought about the relationship, “It still saddens me to think that he’s not, you know, genetically related to me” (Claudia).

#### Preconception strategies

For many women the process of making the child their own began before conception: in their decision making about donation type, their minimization of the donor’s contribution, and/or their normalizing discourses around family diversity.

##### Decisions about donation type

Most women stated a preference for using a donor who was unknown to them at the time of treatment, and many felt that this helped them establish their identity as the child’s only mother by creating more explicit emotional and practical boundaries between their family and the donor, “I think it comes down to that sense of being able to fully own the identity of being the child’s parent” (Eve). Mothers expressed concerns that a known donor could pose a threat to their maternal identity by the donor potentially feeling they had “a stake in [the child]’s upbringing” and by interfering in their parenting by “looking at you or disapproving of the way you were bringing up the child” (Carla). Conversely, for women who used a family member as a donor, the genetic connection between mother and infant was viewed as comforting, “I think both of us thought it would be lovely to have a child who is genetically related to both of us” (Lynda). For these mothers, intrafamily donation enhanced the sense of the child “belonging” in the family through the presence of shared characteristics and physical similarities, “it was great my sister did it because I know [child] will have some of her, my family characteristics, and looks and things like that, so that was important to me” (Lisa).

##### Minimization of the donor’s contribution

For some women, minimization of the donor’s contribution also helped them to claim the child as their own. Often this began before conception but could be used as an ongoing strategy and was seen in mothers’ language when discussing the donation. Some made comparisons with other medical procedures, “It’s only like someone giving you blood” (Christina), or stressed the small size of the donation, “a tiny egg is not really that much of a big deal” (Tanya). A clear distinction was drawn between an egg and a baby: “It was a medical process, it was a donation of tissues essentially, you don’t donate a little mini baby” (Della). For women using known donation, it was important that the donor shared this conceptualization of the egg and was also able to minimize their contribution, “I needed her to say to me that ‘this is all I’m doing, there’s no emotion there, there’s no nothing, it’s not as if I’m giving you my two-year old daughter, I’m literally giving you an egg, that’s it’” (Adele).

##### Normalization of family diversity

Some women used examples of different family relationships and structures to normalize the absence of genetic connections between family members, instead emphasizing family as predicated on shared experiences. Women used examples of half-siblings, step-siblings and adoptive relationships to illustrate the point:

I’ve got a half-brother who’s been brought up separate to me and I haven’t got the same ties with him. . . . I’ve got stepbrothers and sisters. . . . I probably feel a bit closer to them in some respects than I do him because I’ve got memories with them (Rose).

Other women suggested that the concept of family was changing, “I just think families look totally different now” (Aileen), highlighting that assisted reproduction was becoming more common.

For some women, egg donation provoked few concerns and they felt certain prior to conception that it would not make a difference to how they thought or felt about their child, “It wasn’t a big life-changing decision, I was happy to have children whatever way because we wanted a family” (Lily).

#### Importance of pregnancy

For many women, pregnancy was a period during which they had been particularly conscious of the nongenetic relationship. Mothers described feeling uncertain about how they would feel about the baby when it was born, how the baby would feel toward them, and/or what the baby might look like. The extent of these uncertainties varied between women, with several describing “fears” and “panics”, others “worries” and “concerns”, and some never thinking about egg donation while pregnant.

For women who experienced uncertainty about how they would feel about the baby, these feelings centered on whether the child would feel like “my child,” “My only concerns were . . . how was I going to feel about him when he arrived . . . whether I would feel like he was mine” (Florence) and “I was worried that when he was born I would look at him and think he was someone else’s baby” (Hannah). A related concern for some women during pregnancy was whether or not they would be able to bond with the baby, “That’s all I thought about before he arrived, I was just like oh, what if we don’t bond?” (Maggie). Several women had also worried about whether or not the baby would feel able to bond with them, “That was my whole preoccupation . . . would I bond with this baby, would this baby bond with me? My biggest fear was that this baby would think—who the hell are you?” (Leah).

Others described worries around the uncertainty of what, or whom, the baby would look like, expressing concern that they had less control over this aspect than in natural conception:

I didn’t know who she was going to look like. I think when you have a baby naturally . . . like when my niece was born . . . she looks like me, so I think that was the thing I struggled with most . . . if she doesn’t look . . . what am I going to do then? (Gabi).

Despite these concerns, most mothers discussed the central role pregnancy played in conferring ownership of the child, “As far as I’m concerned she was inside of me and so she’s my baby” (Catherine). For many women the idea that during pregnancy they contributed physically to the baby’s development also helped them to feel that the baby was theirs, with mothers often speaking about their role in “nurturing” and “growing” the baby. As one mother said:

Ok, his DNA isn’t mine, but I’ve grown him since he was a 3-day-old embryo so it’s my blood running through his, I’ve fed him, I’ve nurtured him, I’ve grown him, it’s my heartbeat he heard when he was inside me, it’s me he’s going to call mum (Della).

#### Establishing a bond

Establishing a bond with the baby was an important process in the early mother–infant relationship and played a crucial role for many women in enabling them to feel that the child was their own. Although most mothers described feeling a “close”, “strong,” or “loving” bond with their infant, establishing a bond was individual to each mother and occurred at different time points. Several mothers spoke about loving their child from conception or when learning that they were pregnant. Others described bonding during pregnancy, “I felt I really bonded with him when he was in the womb” (Alice). For some women, birth was the point at which they felt they established a connection with their child, “I just loved him unconditionally as soon as he was born” (Nina). For other women, establishing a bond with their child happened during infancy.

Although most women reported no difficulties bonding with their baby, a minority experienced challenges establishing a bond, and attributed this specifically to having used egg donation. For several women, this difficulty was experienced, and resolved, during pregnancy. One mother described finding the first half of her pregnancy challenging:

It was very, very difficult, perhaps for the first good 20 odd weeks. . . . I was angry that I’d managed to get pregnant so easily with someone else’s but not my own, and when people say “oh you must be delighted” it was like. . . . I am, but I’m also very, very sad because I’m still grieving for the fact that I can’t have my own kids (Sophie).

For this mother, feeling able to bond with her baby was not possible until later in the pregnancy when she felt that he had his own personality:

It was later when me and the baby bonded, when he was moving and kicking . . . you could poke him and kick back . . . so you’d have this little game, and that’s when . . . he had a personality, he was a real thing, and that’s when I was like ok, this is my baby.

Several mothers struggled to bond during the early months of infancy and attributed this to concerns about egg donation, despite also acknowledging the difficulty of disentangling the extent to which these problems were compounded by the common challenges of early parenthood:

I really, really did struggle because she was a very colicky baby as well. . . . I had my own lingering doubt about whether I’d done the right thing about using donor eggs and everything . . . certainly in that first few months, I think it did cause a certain amount of emotional separation, on my part. . . . I really couldn’t give myself a hundred percent to her, emotionally. I took care of her, I did what I needed to do, but I didn’t feel like she was my child really. It took a while I think for that feeling to come in (Daisy).

#### Child as wanted

Many mothers’ representations of their baby and of the mother–infant relationship emphasized how wanted the baby was. This was often discussed with reference to how long the couple had waited to have a child and how precious the baby was as a result, thus establishing the central position the child occupied within the family. Babies were often referred to as a “miracle” and “special,” with one mother describing her daughter as “the love of my life. A miracle really. I never thought we were going to have a child so . . . just the best thing that’s ever happened to me” (Megan).

A related narrative used by some mothers, which also served to establish the child within the family, was the belief that had they chosen a different route to parenthood, or been successful on a different treatment cycle, they would not have been a mother to this specific child. For some mothers, this idea was framed in terms of “fate” and that having this child was “meant to be.” In some cases, this fatalistic belief was expressed when mothers talked about the decision to try egg donation; in others, it was used in relation to choosing the right donor. Some described a sense of “knowing” that the treatment would work, “I just knew I was going to get pregnant . . . I just knew it in my heart. It was a very strange feeling” (Gloria), knowing that the pregnancy would be successful, “I felt that this one was going to be different” (Madeline), or knowing that they had ended up with the “right” child, “I’m a big believer in fate, what’s meant to be is meant to be, and we’ve got the right one cos she’s ours” (Katy).

#### Mothers’ influence beyond genetics

One of the ongoing ways in which mothers made the child their own was through emphasizing their contribution to the child’s development through the quality of the mother–infant relationship. Most women spoke about their ability to influence their baby’s personality, development, values and interests through their own behavior and interactions:

I’m hoping she’s quite an extrovert, outgoing little girl, because. . . . I’m not a quiet mum with her. . . . I think I’m quite boisterous with her and quite. . . . I’m all colors of the rainbow with her and I do lots with her. . . . So I do believe you do influence that (Mary).

Highlighting shared mannerisms was another common way in which mothers identified their influence on the child’s development, “His mannerisms are turning out to be the same as me . . . he copies me a lot and I didn’t realize that I gave him a lot of kisses and now he’s started kissing people all the time” (Lucy).

Many mothers proposed a definition of parenting in which commitment and effort mattered more than genetics, *“*[the eggs] could come from somebody who’s educated at Cambridge, a member of Mensa, but if you don’t put in the effort with your children it doesn’t matter” (Maggie). Providing consistent care was believed to be particularly important as it enabled the mother and baby to build a relationship that would sustain into the future and be more influential than a genetic connection. Most mothers were able to see that their efforts were recognized by the baby and could be seen in their baby’s responses. One mother described feeling reassured by her son indicating that he wanted her to be the one who comforted him:

If he wakes up in the night . . . it’s always me that he wants . . . it’s really nice, ‘cos I suppose again . . . with the egg donation thing . . . obviously when sort of those moments happen, it does sort of make you feel, actually yeah . . . it doesn’t matter what the background is or whatever. You’ve been there all the time for him, and he obviously recognizes that (Amanda).

#### Physical resemblance

The extent to which the infant physically resembled family members was discussed in varying ways by mothers and served to either emphasize that the infant “belonged” in the family or could mark them out as different. Most mothers stressed the physical similarities between the baby and father, with the baby frequently described as “the spitting image” or a “mini” version of the father, thus anchoring the child within the family by means of physical resemblance. Although this provided reassurance to some mothers, “she does thankfully look a lot like [partner]” (Katy), for others it also demonstrated to the outside world that the child belonged within the family, “He happens to look very like his father. . . . I think people would have been whispering otherwise” (Abigail).

For many mothers who used identity-release donation, donation type facilitated this process as not knowing what the donor looked like allowed them to interpret the child’s appearance with reference only to the father, “They look like their dad, and I think that’s because we don’t know who the other person is, and we don’t know what they look like” (Tina).

Several women described feeling sadness about not being able to see their own physical characteristics in the child, with this subtly marking the child out as different. Some mothers raised this in a rueful manner, “Sometimes it bothers me cos she doesn’t have, as [partner] says, my fat ankles and stuff like that [laughs]. That’s a good thing, that’s a good thing, or my knees [laughs]. So yeah, sometimes I think that’s a shame” (Katy). In a few cases women became upset when discussing the child’s lack of perceptible family characteristics, as this appeared to represent the loss of something greater and more nebulous, a sense of lost family heritage:

You wonder whether you’ll see parts of other members of your family in your child as well, so. . . . I was imagining . . . my dad died when I was younger and I kind of had an idea that perhaps there’d be part of him . . . [upset tone] . . . just in a look . . . or in the handwriting or just some shadow that’s passed down . . . and you do still think about that yourself . . . what genetic traits and how has that affected my personality from all the previous grandparents, great grandparents and so on, just that history of people behind you as a person (Sophie).

#### Managing reminders of egg donation

For some mothers, the ongoing process of making the child their own involved managing reminders of egg donation. Different factors served as reminders for different women and provoked a range of responses.

##### Comments about the child’s appearance

Some mothers described other people’s comments about the child’s appearance as prompting thoughts about egg donation, “When family who don’t know come out with that he doesn’t look like me, it brings it back then” (Ellen). Some were unconcerned by the comments, others described them as “weird” or “funny”, and several responded directly when people commented on physical similarities between the mother and baby, “I’m like, ‘he doesn’t look like me because he’s an egg donor baby’” (Valerie). Some mothers liked that people commented on physical similarities between themselves and the child, others described experiencing mixed feelings when the child’s physical similarity (or lack thereof) to the mother was mentioned, with several reporting feeling “hurt” by others’ comments.

##### Other reminders

For some mothers, egg donation was brought to mind during medical appointments when they were asked about their family’s medical history. Others mentioned special events, for example Mother’s Day, or the child’s first birthday, acting as reminders. Some mentioned that egg donation was brought to mind in moments when they looked at their baby and thought about how “lucky” they were to have them, or how grateful they were to the donor, “The odd time I’ll look at him and I’ll think oh god, thank god she’s done it” (Nina).

Several mothers mentioned aspects of their baby’s appearance or personality that reminded them about egg donation. For one mother, her daughter’s hair served as a reminder, “She’s got these three curls at the front of her head, so that’s kind of a bit of a reminder about . . . why she’s different to other babies” (Eve). For another mother, knowledge about the donor’s interests and seeing the same interests in her son brought the donation to mind, “Sometimes if I’m in the garden, because she made a comment [in her donor information] about she loves to sit in the garden, and he loves to be outside, and I sort of think, oh that’s nice, the similarity’s there” (Rose).

For several mothers, egg donation was brought to mind when they reflected on whether the child would view everyday parenting decisions through the lens of being donor-conceived. One mother, who also had an older naturally conceived child, described how she had considered this when deciding which bedrooms her children should have:

I’m a bit uneasy about putting [child] in the little one because as she grows up what if she thinks that she’s got the little bedroom so she’s sort of second rate . . . and then I think I’m over-examining this. . . . I think actually if there hadn’t been the IVF with the egg sharer would I be worrying about which bedroom. . . . I’d say look, she’s a baby, she doesn’t need a big bedroom so she’s going in the little one (Steph).

Mothers who mentioned these reminders generally described them as “fleeting”, manageable thoughts, rather than intrusive, persistent, or distressing, with some reporting that they sometimes forgot about egg donation altogether.

#### Finding a place for the donor

Part of the process of making the child their own involved mothers finding a psychological place for the donor with which they felt comfortable. Most mothers felt confident in both their position as the child’s mother and in their relationship with the child, describing the donor in ways that implied little or no sense of threat to the current mother–infant relationship. Mothers often described their “curiosity” or “intrigue” about the donor, particularly in relation to what she looked like, or the origins of the child’s traits or interests, “I sometimes wonder whether they’re academic or sporty or artistic or musical, because he loves music, and I sometimes think ‘I wonder if that’s come from somewhere else’” (Della). These mothers were able to express curiosity about the possible origins of traits, while also being able to manage the uncertainty created by their questions.

Many mothers had thought about and established clear psychological boundaries between the mother–infant dyad and the donor. These boundaries differed between families; for some mothers, boundary management involved allowing very little psychological space for the donor, and for others, it involved reaching a balance between acknowledging the donor and establishing distance. One mother described reading about another family’s regular acknowledgment of their donor and deciding that that model felt too close:

There was one family who called their egg donor Nel, because as the children were growing up, if they ever did things and it wasn’t like mummy or like daddy, they used to say it might be like the Nice Egg Lady . . . and I thought oh, I don’t know whether I want that, to be like talking about them as much as that (Julia).

Another mother described saying a prayer for the donor every evening, designating a place for the donor that gave a low-key though regular acknowledgment of their contribution, yet also establishing clear boundaries between the two families, who were described as leading their own lives:

I think that’s important just to say “thank you” to the kind lady who helped to make [child], as he’s lying snuggling with his little bears, and to look after her and her family . . . that’s just acknowledging her contribution, but without getting hung up on her, she did it, it was a good act, she’s getting on with her life, we’re getting on with bringing up [child] (Grace).

For a minority of women, the donor was perceived as threatening to the mother–infant relationship, with mothers fearing that the donor may want the child as their own. Underlying this fear seemed to be the idea that the donor, having provided genetic material, had a “claim” over the child. These mothers’ narratives were characterized by contradictions and suggestions of the donor wanting the baby. One mother had asked the clinic whether the donor would like to see a photograph of the child, and then realized, “They can’t, which is probably a good thing, because she’d want her. And she can’t have her.” She went on to say:

I think of [the donor] in a grateful way now. I’d like to meet her . . . but. . . . I wouldn’t want to cause her pain. Might be hard for her to see a child that. . . . I kind of want to say is hers. It’s not hers. It’s mine, but it’s still fraught. Still emotionally charged, that whole area (Leah).

Another mother described her lack of genetic relationship as “a bit heart-breaking” and was still struggling at times to feel that her child was entirely hers, imagining instead that the donor was her child’s mother:

There’s certain things I say . . . like when I say something that’s like got “mummy” in the sentence, every so often I’ll think . . . that sounds awful this, oh my god. . . . I’m, it sounds horrible, “I’m not your real mum”. . . . I know I am, but every so often I’ll think. . . . I’ll say “mum” and then I’ll like imagine . . . someone else (Kristina).

## Discussion

The study aimed to explore egg donation mothers’ thoughts and feelings about their developing relationship with their infant. The findings revealed that new egg donation mothers employed a range of strategies across the transition to motherhood that enabled them to establish their identity as the infant’s mother in the developing relationship and feel that the child was their own. This complex and multifaceted process was individual to each woman, with the absence of a genetic relationship between mother and infant varying in significance between mothers. Most egg donation mothers, however, felt secure and confident in their position as the child’s mother by the end of the first year.

The process engaged in by egg donation mothers has some similarities to the process of parental claiming undertaken by adoptive parents during the preadoption period, that is, the emotional and intellectual work carried out in order to gain a sense of entitlement to parent a genetically unrelated child (Sandelowski et al., 1993). Similarities between the two processes include, first, the idea that this emotional work can begin before the child joins the family, and second, that examples of nongenetic close relationships are used as an experiential framework allowing parents to establish that genetic connections are not necessary to form loving relationships or determine parental status. The current study adds to this concept by identifying additional strategies used by egg donation mothers to “claim” the infant as their own, and also highlights the broad and variable timeframe within which this process is undertaken. For mothers in the current sample, making the child their own could begin before conception and continue throughout infancy.

That choice of donation type could help mothers to feel more secure in their identity as the child’s mother has been remarked upon in the donation literature, in cases of both anonymous (Greenfeld & Klock, 2004) and intrafamily donation (Baetens, Devroey, Camus, Van Steirteghem, & Ponjaert-Kristoffersen, 2000). Similarly, several qualitative studies have discussed the significance of pregnancy for egg donation mothers. Although Becker (2000) suggested that a gestational connection usually compensated for the lack of genetic connection, Kirkman (2008) found that this was not always the case. Pregnancy was identified by most women in the current sample as important in helping them to feel that the baby was their own, and as highlighted by Figueiredo et al. (2007), emotional involvement with the infant may be promoted by pregnancy allowing mothers to create an internal world in which to receive the infant. However, for the current sample, pregnancy alone was not sufficient. The finding that some women reported not feeling that the child was their own until later in the first year suggests that for some mothers, conceptualizing the child as one’s own requires additional emotional work (carried out within the context of an actual tangible relationship) over a longer period of time.

The finding that many mothers had been particularly conscious of nongenetic motherhood during pregnancy is consistent with survey data (Van Berkel et al., 2007). The present analysis provides a more detailed account of women’s specific uncertainties and concerns in relation to this issue, the majority of which related to whether a child conceived using egg donation would feel like “my” child. Similar concerns about whether women would feel like a child’s “real” mother, or whether they would feel able to love a genetically unrelated child, have been voiced by women prior to conception with donor eggs, although not specifically during pregnancy (Kirkman, 2008; Stuart-Smith et al., 2012).

Despite several surveys finding that egg donation mothers describe “instant bonding” to their baby at birth (Applegarth & Riddle, 2007; Hertz & Nelson, 2016), the present findings suggest a more individualized and nuanced experience of establishing a bond that occurred at different timepoints for different mothers. Goldberg and colleagues (2013) have argued that adoptive parents may come to parenthood with a “heightened awareness of the bonding process.” The individual nature of the bonding process described by mothers in the present study is in line with findings from both genetically related (Figueiredo et al., 2007) and adoptive parent samples (Goldberg et al., 2013), both of which highlight the variability found in parents’ experiences of the formation of their affectional bonds with their babies. The finding is also in line with the developmental psychology literature that suggests the bonding process involves a psychological component (de Cock et al., 2016) and that these feelings develop dynamically among new parents through engagement with their infant (Koniak-Griffin, Logsdon, Hines-Martin, & Turner, 2006).

Egg donation mothers’ representations of their child as wanted, special, and a miracle echo those found among both donor conception and adoptive parents (Becker, Butler, & Nachtigall, 2005; Daniluk & Hurtig-Mitchell, 2003). Similarly, some women’s descriptions of being destined to become a mother to a specific child, and that their current family constellation was “meant to be,” have also been found in some adoptive parents’ narratives (Daniluk & Hurtig-Mitchell, 2003; Goldberg et al., 2013; Sandelowski et al., 1993). Goldberg et al. (2013) suggested that this fatalistic framing allows parents to claim their adoptive children, and this framing seems to fulfil a similar purpose for some egg donation mothers.

Mothers’ discussions of physical resemblance, both in relation to their emphasis on similarities between the father and child, and to others’ comments about the child’s appearance, highlight the complex, and sometimes uncomfortable, role occupied by physical resemblance within families. Family resemblances are culturally understood as signifying genetic relatedness (Mason, 2008). Becker et al. (2005) found that heterosexual donor-conception parents focused on physical resemblance because they felt vulnerable about it, and because lack of resemblance embodied their sense of loss about the missing genetic connection. They also suggested that resemblance talk serves to situate the child within the family. Although many mothers in the current study did use resemblance talk to anchor the child within the family, and some discussed others’ comments on resemblance serving as reminders of the lack of genetic relationship, this was not necessarily framed in terms of loss, and some mothers were unconcerned by such comments.

The finding that it was possible for some mothers to feel confident in their position as the child’s mother but also to acknowledge ongoing sadness about the lack of genetic connection is in line with Kirkman’s (2008) finding that for some mothers the missing genetic connection remained “a meaningful absence.” However, the findings of the present investigation also suggest that these two feelings are not necessarily incompatible. Again, similarities with adoption can be identified; as Cudmore (2005) suggested, for adoptive parents, the expression of sadness does not inevitably indicate that a loss is unresolved. For some egg donation mothers, the “grief work” associated with infertility may be ongoing after the arrival of the child but can be incorporated into a functional and healthy sense of self, as has been suggested in the transition to adoptive parenthood literature (Brodzinsky, 1997).

Mothers’ strategies could also be considered in light of Kirk’s (1964) theory of adoptive parents’ coping strategies of acknowledgment and rejection of difference. Mothers’ emphasis on the importance of pregnancy, identification of similarities between the father and child, the representation of the child as “fated” to be in the family, or for some, the limited acknowledgment of egg donation, could all be seen as rejection of difference strategies. Kirk (1964) argued that successful rejection of difference helped new adoptive parents to transition into parenthood and feel that the child was their own, and the present findings suggest that some egg donation mothers employed these strategies to fulfil a similar function. Other strategies, however, including consideration of donation type, the acknowledgment of ambivalent feelings regarding the donor, and the acknowledgment of uncertainty about bonding with the child required mothers to engage with the idea that their child is different in some way, and could be viewed as acknowledgment of difference strategies. Brodzinsky and Huffman (1988) suggested that in the case of adoption, rejection of difference strategies may be most beneficial to families at the start of the adoption life cycle, where parents are focused on establishing a secure family unit. The present findings suggest that similar processes may also be occurring in infancy among egg donation mothers, albeit to varying extents between families. Brodzinsky (1987) has argued that a balance between rejection and acknowledgment of difference strategies may be most conducive to positive family functioning in adoptive families; whether these strategies relate to families’ outcomes in egg donation families remains to be established.

That several women felt concerned about the developing relationship and conflicted about the security of their parental role, and some perceived the donor as threatening to the mother–infant relationship, suggests that more research is needed to identify women who may find managing the transition to egg donation parenthood particularly difficult. The finding also highlights that some women may benefit from ongoing psychological support postnatally to adjust to this transition. Clinics in many countries require at least one counseling session prior to treatment with donor eggs (e.g., HFEA, 2019a), but follow-up care is not standard practice (HFEA, 2019a). It is possible that mothers who felt more comfortable with their adjustment to egg donation motherhood may have been more likely to participate in the current study, and as such, women who were struggling with this process, although present, may be underrepresented in the current sample.

With regard to the recruitment of participants, no information is available about the families who chose not to pass on their contact details to the researchers, and little is known about families who did so and then declined to participate. All participants were told that clinics would not be informed about their study participation, so to request information from clinics about nonresponders would have compromised the anonymity of participants. As a result, it is not possible to know whether participating families differed from those who chose not to.

A small, but growing, body of literature has explored trends in parents’ intentions and decisions about disclosure of egg donation conception to their children (Readings et al., 2011; Söderström-Anttila et al., 2010), and how these decisions relate to child adjustment (Golombok et al., 2013). It is possible that mothers who perceive the donor as more threatening may be less likely to tell the child about their method of conception, and thus future work with mothers of older children should also look to explore the relations between mothers’ feelings about egg donation motherhood, their representations of the donor, and their disclosure decisions.

Egg donation mothers’ representations of the mother–infant relationship revealed complex and ambivalent feelings about the role of the nongenetic connection in the developing relationship. Although some of these themes were specific to donor conception (e.g., finding a psychological place for the donor), others were not. For example, mothers’ concerns about whether they would bond with their baby, questions about what their baby would be like, and thoughts about their influence on the child’s development are shared by many parents (Leerkes & Crockenberg, 2002; Redshaw & Martin, 2013). Despite the commonality of these experiences, egg donation mothers tended not to discuss them in these terms and instead viewed these issues through the lens of genetic relatedness. This emphasis on genetic connection is perhaps unsurprising given that mothers are raising children in what has been described as the “age of genetic essentialism” (Freeman, 2014). A more open discussion about some of the similar experiences of the transition to parenthood for mothers in different family types may go some way to reducing the stigma felt by some egg donation families.

With regard to implications for professionals working with women considering egg donation or egg donation mothers, acknowledging and normalizing the highly individualized nature of the process of claiming a child as one’s own seems valuable. For example, knowing that concerns about whether the child would feel like their own were common to many pregnant egg donation mothers, or that most mothers felt that the baby was their own by the end of the first year, may prove reassuring to both women considering treatment and pregnant patients. Similarly, normalization of the individual nature of this process may be valuable to egg donation mothers who are finding that feeling that the child is their own has taken longer than expected. Within clinical settings and among egg donation parents it also seems important to normalize the idea that mothers can express ambivalence and uncertainties about the nongenetic relationship with their infant, but still feel confident and secure in their identity as the child’s mother.
